# Secretory A immunoglobulin, total proteins and salivary flow in Recurrent Aphthous Ulceration

**DOI:** 10.1016/S1808-8694(15)30075-6

**Published:** 2015-10-19

**Authors:** Kennedy de Oliveira Martinez, Lauro Lúcio Mendes, José Bento Alves

**Affiliations:** a M.S. Professor of Morphologic Sciences - University of Uberaba; b PhD. Professor. Department of Restorative Dentistry - Dentistry School - UFMG; c PhD. Research, Postgraduate Programs and Extension Programs Dean - University of Uberaba. Universidade de Uberaba, Uberaba MG

**Keywords:** salivary immunoglobulin, saliva, recurrent aphthous ulceration

## Abstract

Clinical and experimental study. Introduction. Of debatable etiology, Recurrent Aphthous Ulcerations (R.A.U), is most of the time considered an immunological deficiency. **Aim:** The aim of this paper is to review the literature and clinical investigations regarding IgA-s, total proteins salivary concentration and basal salivary flow of patients with R.A.U. during activity and quiescence. **Methodology.** Nephelometry was used to measure salivary IgA-s; Pyrogallol red was used for total salivary proteins and the gravimetrical analysis for salivary flow measurement. **Results.** Results demonstrated a significant increase in salivary IgA-s in active lesions in relation to quiescence. On the other hand, protein concentration rates were similar in both periods. **Conclusion.** Salivary IgA-s can be used as a parameter to study the immune status of the oral mucosa.

## INTRODUCTION

Hipocrates (460-370 a.C.) was the one who, for the first time, employed the word “aphtha” (aphtodea)^1^, coming from the Greek word a p t e i n (ARTEIV), translated as “set fire”^2^. Until today, the etiology of recurrent aphthous ulcerations (RAU) remains unknown, although hints of its etiologic basis lay on genetic susceptibility, infectious agents and alterations in immune mechanics.

Now, the mouth mucosal protection system is well known, and gathers a large number of substances responsible for its homeostasis^3^.

The most important humoral mediator for mucosal immunity is secretory IgA^4^, ^5^, for cooperating with a number of protection mechanisms^6^, for presenting greater resistance to the proteolytic degradation caused by other immuneglobulins^7^ and for being especially located in the digestive and respiratory tracts, which are in close contact with the environment^8^, preventing the uptake of a large amount of antigens and precluding an overload in the immune system^9^.

The secretory A immunoglobulins present in saliva are excellent indicators of the oral mucosa immune status^10^, ^11^, ^12^, nonetheless, clinical-laboratorial comparisons of recurrent aphthous ulcerations and the local protecting mechanisms involved indicate the need for further studies, having in mind the scarce number of papers published and contradictory results.

## MATERIALS AND METHODS

The present investigation, approved by the Research Ethics Committee of the Federal University of Vales do Jequitinhonha e Mucuri Dentistry School, counted on the participation of twenty patients with prior history of recurrent aphthous ulcerations, seen in the department of Stomatology of the aforementioned institution. The patients were selected regardless of gender, age, race or socio-economical situation, who matched the inclusion criteria, such as no chronic systemic diseases, drug therapy in the week prior to the exam and recent dental treatment. All patients were informed on the procedures before entering the research project, and signed an informed consent form.

In order to obtain a sample of total saliva, the patients were instructed as not to eat or drink (except water) for one hour and mouth hygiene at least two hours before the procedure. Mouth washing with pure water was carried out right before harvesting the samples (between 8 and 10 am). Sampling time was never above 15 minutes.

Total spontaneous saliva that collected on the oral cavity floor was harvested with disposable sterilized Pasteur-type pipettes. After harvesting, 1.5ml of each sample was placed in coded Eppendorf tubes and centrifuged afterwards, in order to reduce bubble and foam, and stored in a refrigerated system (freezer at -20ºC). All patients participated in two times during the research. The first was when they had active aphthous lesion, and the second was when they did not have the lesion (quiescent period).

### Biochemical Analysis

Nefphelometry was used in order to quantitatively assess salivary IgAs (Behring Nephelometer II, Behring, USA) with calibrators and reagents supplied by the manufacturer. In order to assess total proteins, we used the Pyrogallol red (Sensiprotò) and reading carried out by a semi-automatic analyzer (550 Express from CIBA - Corningò).

After harvesting, we carried out spontaneous salivary flow measures (flow at rest) at the times of active lesion and at quiescence. During this evaluation, all patients remained seated and relaxed, with their heads down and lips slightly open in such a way as to let saliva drool passively over the lower lip and be collected in a graduated beaker of known weight. 5 minutes was taken for each sampling. Salivary flow was determined by gravimetric analysis^13^, ^14^.

### Statistical Analysis

Comparisons were made between male and female patients for each one of the time points (with and without aphtha) in relation to the variables’ measures: salivary flow, salivary IgA-s and total salivary proteins concentration, using the Kruskal-Wallis test. This test compares two or more independent samples in relation to a variable of interest.

Aiming at comparing samples (at the time points with and without aphthae) by gender, in relation to the variables’ measures: salivary flow rate, IgA-s and total salivary proteins concentration (regardless of gender in relation to salivary flow and salivary IgA-s), we used the Wilcoxon non-parametric test of paired samples.

All results were considered significant for a significance likelihood below 5% (p<0.05) thus, yielding at least 95% of confidence in the conclusions presented.

## RESULTS

Of the selected patients, 12 (60%) were females and 8 (40%) were males, within an age range between 07 and 54 years, with a prevalence in the 3rd decade of life (from 21 to 30 years). As related to age, there were no significant differences (p> 0.05) gender wise; in other words, the ages of these two groups of patients were similar.

The lesions varied between 1 and 4mm in diameter and prevailed on the tongue, gingival-labial notch and upper lip, with an average number of 2 (two) lesions per patient.

Predisposing factors reported were: stress, food, gastrointestinal disorders, menstrual cycle, family history and in some it was not possible to notice any association with predisposing factors (no apparent cause).

Results regarding patients’ salivary flow and IgA-s level evaluations at the time points with and without aphtha, regardless of gender, are presented on (Table 1). Notice that there were no statistically significant difference between salivary flow mean values of the patients at the two time points - active lesion and quiescence. As to total protein values at the two time points, gender-related, we noticed that for males the values were statistically higher (Table 2). On the other hand, the total proteins concentration was different considering the two time points (activity and quiescence) in recurrent aphthous ulcerations (Table 3). The levels of salivary IgA-s in the results proved to be significantly increased at the time of activities of this lesion, when compared to results at quiescent periods (Table 3).

**Table 1:** Comparative and descriptive analysis of salivary flow and salivary IgA among patients with and without aphthous lesions, regardless of gender.

Descriptive measures (level)								
Variable	Group	n	Min.	Max.	Median	Average	d.p.	p
Salivary flow	With aphtha	20	0.18	0.52	0.33	0.34	0.09	0.575
(ml/min)	Without aphtha	20	0.15	0.50	0.38	0.34	0.10	CA = SA
Salivary IgA (mg/dl)	With aphtha	20	3.2	17.4	7.5	8.2	3.4	0.001
	Without aphtha	20	3.0	12.0	5.4	6.0	2.3	W/A > W/out A

Note: The p value refers to the Paired Wilcoxon Test

Legend: W/A: with aphtha; and W/out A: without aphtha

**Table 2:** Descriptive and comparative analysis of the salivary flow analysis, salivary IgA and total salivary proteins level between genders in relation to patients with aphthae.

Descriptive measures (level)								
Variable Patients with aphtha	Gender	n	Min.	Max.	Median	Mean	s.d.	p
Salivary Flow	Male	8	0.18	0.50	0.31	0.32	0.10	0.315
Rate (ml/min)	Female	12	0.24	0.52	0.37	0.36	0.08	M = F
Salivary IgA (mg/dl)	Male	8	3.2	13.0	7.2	7.6	3.0	0.643
	Female	12	4.3	17.4	7.8	8.6	3.8	M = F
Total salivary	Male	8	36.0	81.1	48.4	51.6	13.7	0.049
proteins (mg/dl)	Female	12	24.8	85.0	41.6	42.2	15.5	M > F

Note: p value reflects the Kruskal-Wallis test

Legend: Gender: M: Male and F: Female

**Table 3:** Descriptive and comparative analysis of salivary flow, salivary IgA and total salivary protein concentration between the genders, in relation to the patients without aphthae.

Descriptive measures (level)								
Variable	Gender	n	Min.	Max.	Median	Mean	s.d.	p
Patients without aphthae								
Salivary	Male	8	0.17	0.50	0.34	0.32	0.12	0.561
Flow (ml/min)	Female	12	0.15	0.45	0.38	0.35	0.09	M = F
Salivary IgA (mg/dl)	Male	8	3.0	12.0	6.7	6.6	2.7	0.263
Total salivary	Male	8	39.7	81.1	65.9	51.6	12.8	0.014
proteins (mg/dl)	Female	12	22.9	85.0	45.9	42.2	10.1	M > F

Note: p value reflects the Kruskal-Wallis test

Legend: Gender: M: Male and F: Female

## DISCUSSION

The word “aphtha” has been used both by health care professionals as by laymen in order to call any oral lesion characterized by pain similar to those caused by a burn^2^.

In this investigation, the term recurrent aphthous ulcerations (RAU) was employed in order to avoid confusions with other similar entities.

The diagnosis of RAU is purely clinical, considering its semiologic and evolution characteristics^15^. The histopathology of these ulcerations does not show specificity, it was used mainly to rule out the non-aphthous lesions^7^, since it represents the histopathology of a non-specific inflammatory process^16^.

Recurrent aphthous ulcerations are usually classified as: major, minor and herpetiform, according to their clinical manifestations such as number of lesions, location and severity^17^. In our series, the most prevalent type was the minor, with onset average time of 5 days.

Spontaneous saliva was preferred over the stimulated flow, since there are already data in the literature about the “flow at rest”, and because it is more reliable to represent the physiology of oral secretions without the interference of external factors.

Despite the small amount, organic substances are molecules responsible for a major part of the saliva’s biologic functions^18^; they are part of the immune system and may, to some extent, operate independently of the general immune system^4^.

Secretory immunoglobulin A (IgA -s) is an important parameter to assess the autoimmune status of the mucosa, with the advantage of being measurable by non-invasive methods and without patient discomfort^19^, ^20^. This is the major class of immunoglobulin found in mucous secretions, and is responsible for a barrier against a number of infectious, environmental allergens and carcinogenic substances^19^, and it also aids in many inborn protection mechanisms^8^.

IgA represents the second most abundant immunoglobulin in the human serum and prevails in the saliva in its dimeric form (IgA-s) which better resists proteolysis in environments such as the mouth^21^.

IgA deficiency is the most common immune humoral defect in humans, and for the most part, causes gastrointestinal and respiratory infections^22^, ^23^.

If immunity has some regulating effect on the development of recurrent ulcers, what is expected is that IgA-s be the immunoglobulin that provides protection, considering its role in other body parts.

In this study, we aimed at investigating IgA-s concentrations and that of total proteins found in the total spontaneous saliva and check to see if they differ during the times of quiescence and exacerbation of recurrent aphthae.

As a means to check salivary flow variations, which could influence IgA-s and total protein levels, we used total saliva at rest flow check. Our results showed that there were no statistically significant differences between flow measures at the times of active lesion and acquiescence (table 1). Similar results were obtained from patients with RAU when compared to individuals without prior history of such lesions^24^.

We also did not see statistically significant differences between total protein concentrations in patients with and without aphthae.

Now, total protein concentrations did not vary when the patients were compared with and without lesions (Table 3). Spontaneous and stimulated total saliva flow sample analyses showed that salivary flow was lower and total protein concentrations were higher in women when compared to men^13^. in the present investigation, when we compared total proteins we found higher concentrations (p < 0.05) at the two time points (With and Without aphthae) in men. On the other hand, salivary flow mean values did not present significant difference between genders (according to Table 2). It is interesting to notice that, although men presented higher concentrations of total proteins when compared to women, we noticed that both for men and women, total protein concentrations were similar for both time points: with and without aphthae. There were no statistically significant differences (p>0.05) between these two time endpoints (Tables 4 and 5).

**Table 4:** Descriptive and comparative analysis of salivary flow, Salivary IgA and total salivary proteins between patients with and without aphthae, insofar as males are concerned.

Descriptive measures (level)								
Variable Male patients	Group	n	Min.	Max.	Median	Mean value	s.d.	P
Salivary	With aphthae	8	0.18	0.50	0.31	0.32	0.10	0.944
flow (ml/min)	Without aphthae	8	0.17	0.50	0.34	0.32	0.12	CA = SA
Salivary IgA (mg/dl)	With aphthae	8	3.2	13.0	7.2	7.6	3.0	0.021
	Without aphtha	8	3.0	12.0	6.7	6.6	2.7	CA >gt;gt; SA
Total Salivary	With aphthae	8	36.0	81.1	48.4	51.6	13.7	0.183
Proteins (mg/dl)	Without aphthae	8	39.7	81.1	65.9	51.6	12.8	CA = SA

Note: p value is related to the Wilcoxon’s test

Legend: CA means with aphtha and SA means without aphtha

**Table 5:** Descriptive and comparative analysis of salivary flow, Salivary IgA and total salivary proteins level between patients with and without aphthae, insofar as women are concerned.

Descriptive measures (level)								
Variable Female patients	Group	n	Min.	Max.	Median	Mean value	s.d.	P
Salivary	With aphtha	12	0.24	0.52	0.37	0.36	0.08	0.347
Flow	Without aphtha	12	0.15	0.45	0.38	0.35	0.09	CA = SA
Salivary IgA	With aphtha	12	4.3	17.4	7.8	8.6	3.8	0.011
	Without aphtha	12	3.5	10.2	5.1	5.6	2.1	CA > SA
Total salivary	With aphtha	12	24.8	85.0	41.6	42.2	15.5	0.255
proteins	Without aphtha	12	22.9	85.0	45.9	42.2	10.1	CA = SA

Note: p value is related to the Wilcoxon’s test

Legend: CA means with aphtha and SA means without aphtha

As to the salivary IgA-s values, there was a statistically significant difference (p<0.05) in the values, noticeably higher during lesion exacerbation when compared to inter-crisis periods (without lesions), as described on Table 1. Moreover, Table 3 corroborates these results, that is, both for males and females; the salivary flow variable in patients with and without aphthae does not differ statistically, while salivary IgA-s levels were statistically higher in patients with aphthae. These data are similar to those found in individuals with aphtha and those without prior history of this lesion^25^, ^26^.

A study carried out on salivary IgA-s levels in patients with RAU at the times of incipient and acute lesion, regression and complete cure, showed an increase in IgA-s at the acute period, and its reduction in periods of regression and cure, and for the women in the group, very discrepant results were noticed, in some of them salivary IgA-s levels were influenced by menstrual cycle and in others there were no such association^27^. This leads to this unreliable correlation between IgA-s salivary levels and the menstrual period, even in those with association between the menstrual cycle and recurrent aphthous ulcerations^28^. Our series had only one woman (of the 12 females in the group) who reported having aphthous ulcerations at the time of menstruation.

## CONCLUSION

Our results suggest a strong correlation between IgA-s and the lesion mechanisms in recurrent aphthous ulcerations, and this may be used as a parameter to assess the mucosal immune status.
